# Perception, Knowledge and Behaviors Related to Typhoon: A Cross Sectional Study among Rural Residents in Zhejiang, China

**DOI:** 10.3390/ijerph14050492

**Published:** 2017-05-06

**Authors:** Wenchao Zhang, Wei Wang, Junfen Lin, Ying Zhang, Xiaopeng Shang, Xin Wang, Meilin Huang, Shike Liu, Wei Ma

**Affiliations:** 1 Department of Epidemiology, School of Public Health, Shandong University, Jinan 250012, Shandong, China; zwchao92@163.com; 2 Zhejiang Provincial Center for Disease Control and Prevention, Hangzhou 310051, Zhejiang, China; shandawangwei@163.com (W.W.); zjlinjunfen@163.com (J.L.); shxiaopeng112@163.com (X.S.); 3 School of Public Health, University of Sydney, Sydney 2006, Australia; ying.zhang@sydney.edu.au; 4 Bureau of human resources and social security of Fengtai District of Beijing, Beijing 100073, China; wangxin0730@yeah.net; 5 Ninghai County Center for Disease Control and Prevention, Ningbo 315600, Zhejiang, China; hmlzzx@163.com (M.H.); shikeliu@163.com (S.L.); 6 Shandong University Climate Change and Health Center, Jinan 250012, Shandong, China

**Keywords:** typhoon disaster, risk perception, attitude, knowledge, behavior, rural residents

## Abstract

(1) The objective of this study was to assess the risk perceptions, attitudes, knowledge, and behaviors related to typhoon among rural residents in Zhejiang province of China. A cross-sectional study was conducted among rural residents in Zhejiang province, China. Information was collected from 659 participants using a structured questionnaire. Univariate analysis and multivariable analysis were used to analyze the data. Participants were most concerned about property damage, followed by their health and life. Television, short message service (SMS), relatives and friends were the most common information sources. Most people had not been educated with disaster prevention measures. The complementary log–log (CLL) model showed that understanding typhoon warning signal, preparation time, risk perception of health damage and life threat, and fears of typhoon were independent predictors of adoption of coping behaviors. We found that: 1. Residents’ risk perception of health and life threat caused by typhoon is inadequate; 2. There is a gap between residents’ cognition or knowledge and behavior in rural areas; 3. The government should further make strategies to develop educational activities, in order to eliminate the gap and improve the ability of preparing for typhoon among rural residents.

## 1. Introduction

Typhoons are considered to be extremely devastating natural hazards worldwide. There are seven to eight typhoons on average landing on the coastline areas of southeastern China each year, which makes China one of the countries that were hit most frequently by typhoons [1,2]. Between 1980 and 2012, there were nearly 300 typhoon landfalls, and 61% of the landmass of China was affected by typhoons at various degree [3]. As a major natural disaster in China, typhoons have caused enormous property and human losses [4,5]. The influence of typhoon disasters permeated through all aspects of people’s activities who live in the typhoon landing regions. Typhoons do not merely destroy infrastructures and facilities, but also cause serious threats to people’s health and life [6]. In addition to immediate injuries and deaths caused by the disaster, typhoon severely influences public health [7,8].

Studies have reported that the impact of typhoon disaster depends on the intensity and strengths of the typhoon, which is also linked with the precautionary concerns and knowledge of local residents [9]. Lack of attention on disaster warnings might lead to significant damages and bring risks of typhoon related injuries even facing a low-risk typhoon, which suggests a need to pay attention to typhoon disaster prevention, regardless of the intensity of storms [10,11].

Knowledge of the public’s awareness about risks from natural disaster is of vital importance to effective risk communication strategies [12,13]. In recent years, many studies put an increasing emphasis on the public’s knowledge and risk perception of natural disaster. Studies in China suggests that risk perception of disaster among residents in typhoon-prone area is higher than other people. In addition, residents’ coping behaviors were positively associated with risk perception and knowledge of preparedness [9,14]. In southern Thailand, a study found that people with previous experience have high risk perceptions which were expressed as a heightened fear of a typhoon and a tendency to take threatened disasters seriously [15]. Residents’ attitudes and risk perception are correlated with knowledge, which have an influence their coping behaviors [16]. Study also suggests that many people are aware of coming cyclones, but are unaware of their intensity and where they would make landfall, and there is also inadequate knowledge on how to prepare for a cyclone [17].

Previous studies have indicated that residents’ awareness and coping behaviors of typhoon disaster was related to their socio-demographic characteristics, especially educational status and economic conditions [18,19]. Rural residents are more vulnerable to typhoon disaster because they live in a comparatively less-developed and impoverished areas, their major concern is the impact on their livelihood, which may lead to insufficient protective measures for themselves [20]. Study also shows risk perception and knowledge of typhoon among rural residents is lower than urban ones, which make them vulnerable [21,22]. Hence, a better understanding of rural residents’ risk perception and knowledge was conducive for government to develop customized strategy. There is also a need to explore the unknown factors influencing rural residents’ coping behaviors.

It has been reported that among rural residents, the KAP-gap (knowledge–attitudes–practice gap) hampers their ability to takes valid adaptive measures [23]. People may have some knowledge about the coming disaster but a lower risk perception could influence subsequent likelihood to adopt actions for preparation [24]. Most people in disaster-prone regions know they should make preparations, but research suggests that very few actually do. In some cases, while residents generally felt prepared for a typhoon disaster, it has been found that there was in fact still insufficient preparation [25]. Even people who had experienced typhoon many times might not take adequate typhoon preparation actions, which increased population vulnerability [26]. A study in Iran showed that the knowledge of people regarding disaster preparedness was encouraging, but the translation of knowledge into effective attitudes and appropriate practices was weak [27]. There is a gap between residents’ perception or knowledge and behaviors. This research project further explores the extent of public perception and knowledge of typhoon disaster, as well as the ability of coping behaviors in a rural area exposed to typhoons.

Among the southeastern regions of China, Zhejiang province is one of the very high-risk provinces most frequently affected by typhoons [28]. Super Typhoon “Chan-hom” landed in the middle coast of Zhejiang Province in July 2015, affecting 1.9 million people with a direct economic loss of almost 6 billion RMB, forcing 1.06 million people to evacuate [29]. The objective of this study was to investigate local rural residents’ risk perceptions, attitudes, knowledge, and behaviors related to typhoon. We have also examined the factors influencing participants’ coping behaviors before typhoon. This study will provide useful information for policy makers and other stakeholders to reduce losses from typhoon disasters in rural residents. The study also provides the opportunity to test theories that guide the disaster risk management decision making in typhoon affected regions, and add to the body of knowledge on the subject.

## 2. Materials and Methods

### 2.1. Study Design and Setting

A cross-sectional study was conducted in July 2015, 10 days after the typhoon “Chan-hom” landing on the southeast coastal area of Zhejiang. Ninghai, one of the counties affected by “Chan-hom” was selected as study site. Ninghai is a county with multiple typhoons every year and has contingency plans for typhoon disasters. Cluster sampling was used to choose three villages in Ninghai. The population of the three villages ranged from 1400 to 1900 and the maximum distance between these villages and coastlines is no more than 10 km. We calculated the sample size for this study according to the formula of survey sample size calculation [30], as well as reviewed the relevant articles and the assumption that 60% of people might adopt adaptive behaviors during typhoon. To estimate this proportion with a 95% confidence interval, 522 participants are required. To allow for a 10% non-response and for 1% missing data, the lowest required sample size is 587.

### 2.2. Study Participants

The targeted study population was local residents of the three villages. The criteria for the participants to be included were: (1) local resident or had lived in the village for at least one year; (2) in the village during the typhoon; and (3) 15 to 74 years old. All eligible residents were invited to participate in the study. Migrant workers, people with mental diseases, and unconscious persons were not included.

### 2.3. Data Collection

A structured questionnaire was developed after a review of the literature in combination with consultations with local CDC experts who are familiar with the situation of local residents and typhoon. The questionnaire collected the following information: (1) demographic characteristics, such as gender, age, race, educational level, occupation and marital status; (2) source of typhoon warning information and risk perceptions of typhoon (all perception questions had closed-ended responses using different five-point Likert-type answers, ranging from rank 1 to rank 5, with a higher score indicating a higher perception level); (3) attitude towards typhoon disaster prevention; (4) knowledge and individual behaviors to cope with typhoon disasters, including a question about awareness of typhoon warning signal; (5) disaster insurance and government’s measures on evacuation.

Interviewers were trained staff at the local Centers for Disease Control (CDC) and graduate students from Shandong University, who were familiar with field interviews and received training before the survey. A quality control person was assigned to each of the seven groups of interviewers. All information was collected by face-to-face interviews at participants’ homes. Thirty-three people refused to participate in our study. The main reason being the perception of receiving no benefit from the study and/or a waste of their time. A total of 683 participants were interviewed with a response rate of 95.4%. There were 24 (3.5%) questionnaires having too much missing information and errors, which were excluded before being entered into the computers. We included 659 valid questionnaires in our analysis.

### 2.4. Statistical Analysis

Data were entered into Epidata version 3.1. Statistical analysis was conducted with the Statistical Package for Social Sciences (SPSS) version 21. Descriptive statistics were used to describe respondents’ demographic characteristics as well as their perceptions, attitudes and behaviors. Chi square or Fisher’s exact texts were used to test for the relationships between demographic variables and other categorical variables. The mean and standard deviation (SD) was used to describe the score of risk perception. Factors with significant associations (*p* < 0.15) from the univariate analysis were then analyzed by complementary log–log (CLL) regression for the reason that the Yes/No responses of dependent variable were asymmetrically distributed [31,32], to further explore how risk perceptions and attitudes may influence residents’ coping behaviors (Adopt or Not Adopt: there are many questions regarding coping measures in our questionnaire. If participants did not have any behavioral change in response to the typhoon, then the individual was defined as not adopting coping behaviors).

### 2.5. Ethical Considerations

The Ethics Review Committee (ERC) of Public Health at Shandong University has reviewed and approved the proposed use of human subjects in this survey (No. 20120501). Written informed consent was obtained from each participant (where participants are children, their parents) after they were informed that their participation was voluntary and their refusal to participate would have no negative consequences. All data were kept anonymous and confidential throughout the study.

## 3. Results

### 3.1. Demographic Characteristics of Participants

The mean age of participants was 48.9 years with a standard deviation (SD) of 14.0 years. The majority of respondents was in their middle age or older (45 years old and above, 64.3%) and currently married (87.4%). As the study was conducted in coastal rural villages, almost all the respondents (98.8%) had a rural household registration and most participants were farmers (71.0%). Over half of the participants (55.1%) had primary and lower level of education including 30.1% were illiterate, 40.2% had secondary education, only 4.7% had post-secondary education or higher. The majority (88.4%) of the illiterate group was over 50 years old. There was no significant relation between adopting behaviors and gender, age, education, or marital status. (Table 1)

### 3.2. Sources of Typhoon Information

Television media was the most commonly mentioned way (83.5%) to get information before a typhoon, followed by short message service (SMS) (32.7%), and relatives/friends (27.8%). As for the most favorable way, people were inclined to television media (76.8%), SMS (43.9%) and house-to-house notification (36.0%) (Table 2). There was a statistically significant association between age or education level and the means to get information. People aged 50 and over (χ^2^ = 14.087, *p* < 0.001) and illiterate (χ^2^ = 3.848, *p* = 0.033) preferred to house-to-house notification, while people who were less than 50 years old (χ^2^ = 70.070, *p* < 0.001) and educated (χ^2^ = 72.691, *p* < 0.001) preferred SMS. The average time that respondents were informed with typhoon warning information in advance was 2.8 days (median 3, SD 1.1), with a range from one to five days.

### 3.3. Attitude to Typhoon Disaster Prevention and Education

Table 2 also shows that 93.3% respondents pay much attention to typhoon disaster prevention. However, the majority (68.9%) had not been educated formally with how to cope with typhoon disasters beforehand. Among the 659 respondents, 89.7% thought typhoon disaster prevention education was necessary, among which 18.1% indicated a strong need; 83.2% indicated that they would attend a formal training. Regarding the governmental measures, e.g., evacuation or temporary shelters, 94.1% of the participants would welcome such support, with 30.3% showed a strong support. Results of cross tabulation analysis between demographic groups and information sources and attitude to typhoon-related disaster prevention can be found in the supplement.

### 3.4. Risk Perception of Residents before Typhoon

Table 3 shows participants’ risk perception of typhoon before its landfall with regard to disaster impact, property damage, health damage, life threat, and degree of fear. Over three-quarters thought that the impact of typhoon disaster was big or very big (scored 4 or 5 out of maximum 5). There were 76.5% respondents who thought typhoon could cause property damage, among which less than one fifth of the respondents thought it would be highly likely to cause property damage. However, merely 50.7% of respondents believed that typhoon could pose a threat to their health and 42.2% believed the typhoon could pose a threat to life. Meanwhile, the score also showed that residents’ risk perception about the impact of typhoon (Mean = 3.89, SD = 0.886) and property damage (Mean = 3.84, SD = 0.837) were high, while that of health damage (Mean = 3.21, SD = 1.003) and life threats (Mean = 3.01, SD = 1.019) were not enough. T test shows that there was no significant difference of the four items above between male and female. A Cochran–Armitage trend test was used to test the mean score across educational levels, which showed no significant difference. Women were more scared of the typhoon than men (χ^2^ = 31.293, *p* < 0.001), so were people over 50 years old (χ^2^ = 20.182, *p* < 0.001).

### 3.5. The Cognition, Knowledge and Behaviors of Typhoon Disaster Prevention

Most participants (74.9%) claimed that they knew how to prepare for the typhoon disaster, with the leading measures including: shutting the doors and windows (93.0%), keeping a watchful eye on typhoon warning (54.8%), preparing emergency articles include foods and drugs (54.0%), leaving the dangerous area (53.0%), keeping away from low-lying areas (48.2%), and checking the electrical circuit facilities (44.3%). Most participants knew that shutting the doors and windows could reduce losses, however, the awareness rate of other measures was not high, and the highest rate was 54.8%. The awareness rate of typhoon warning signal among our respondents was only 11.9%. As for coping behaviors taken, 82.9% participants claimed that they adopted measures before and during typhoon. However, this claim was inconsistent with the responses to the questions about the specific adoptive measures taken, only 434 (65.9%) stopped all outdoor activities, 289 (43.9%) repaired and reinforced their house, 277 (42.0%) stored foods and water. Of the participants, 17.1% did not adopt measures at all for the following reasons: (1) Did not know how to adopt measures; (2) Unnecessary to adopt measures; (3) No time to adopt measures. Among those respondents who adopted coping behaviors during typhoon, 94.4% thought it effective. The majority (73.3%) of the participants did not purchase disaster insurance, especially property insurance and agricultural meteorological disasters insurance [33], mainly for the following reasons: (1) Did not know of such kinds of insurances (73.0%); (2) Not necessary to purchase (14.6%); (3) Financial reasons (10.8%). However, 77.8% thought it would be necessary to purchase a disaster insurance. (Table 4)

### 3.6. Factors Influencing Residents’ Coping Behaviors—Multivariable Analysis

In the univariate analysis, understanding typhoon warning signal, preparation time, perception of property damage, perception of health and life damage, fears of typhoon and attention were found to be significant factors (at 0.15 level) that affected the adaptation of coping behaviors. The factors were then included in the CLL model in the multivariable analysis. The results showed that knowing about the typhoon warning signal, preparation time, perception of health and life damage, and fears of typhoon were significant predictors of the adoption of coping behaviors while perception of property damage and attention were not statistically significant. Respondents with a better awareness of the typhoon warning signal (odds ratio [OR] = 1.96, 95% confidence interval [CI]: 1.30–2.95), more days informed before typhoon (OR = 1.36, 95% CI: 1.12–1.64), and higher fear of typhoon (OR = 1.67, 95% CI: 1.28–2.17) were found to be more likely to adopt coping behaviors (Table 5).

## 4. Discussion

In this study, we investigated the risk perception, attitude, knowledge, and behaviors related to typhoon among vulnerable residents in Zhejiang province which will be useful in preparing for future catastrophic events. Our results show that respondents had a high-risk perception of property damage, while the risk perception of health damage and life threat caused by typhoon was not enough. Majority of participants did not receive any formal education about typhoon disaster prevention prior. This could be a factor in lack of appropriate actual coping behavior. Although the majority. Although majority of them claimed they paid attention to typhoon disaster prevention and knew how to adopt measures, the actual coping behavior before and during typhoon was inadequate (refer to Table 4).

In our study, most residents identified television as the main ways to get information before typhoon (83.5%), the result was similar with previous studies in China [14,28], Florida [26], and New Jersey [34]. TV is a major and convenient source of information among rural residents. A good use of TV to release information by the government plays a major role in helping people to notice the typhoon warnings. As for the most favorable way to get information, the leading three methods were TV media, SMS and house-to-house notification. This result was consistent with a cross-sectional study in Bangladesh, which indicated that mass media and communication between neighbors were important information source [18]. The young people were prone to choose SMS, while the elder people choose house-to-house notification. Considering the age structure of rural residents, we suggest expanding the door-to-door notification by village committee or local government in typhoon-prone rural areas.

With regard to the risk perception before typhoons, participants in our study showed a high concern on property damage, while an insufficient concern on health damage and life threat regardless of residents' gender, age or educational level. This result was similar with the previous study in New Jersey which indicated that the greatest concerns of residents before the typhoon was possessions and property damage, while during and after the disaster, their major concerns were personal health [34]. In our study, only about half of participants affirmatively express that typhoon might pose threat to their health (50.7%) or life (42.2%), and less than half of the participants (37.4%) expressed a fear of typhoons. Similarly, according to a survey in 2009, 45% participants did not realize that they were vulnerable to disaster [35].

Our results showed that there was no difference between residents’ risk perception, coping behaviors and their socio-demographic characteristics which is inconsistent with previous studies [14,18]. The main reason could be that we conducted our survey in rural areas only, the majority of respondents were in their middle age or older, and over half of the participants had primary and lower level of education, including the 30.1% that were illiterate. In addition, we investigated three adjacent villages where residents may share similar experience about typhoon.

Residents have paid great attention to typhoon disaster prevention, and most residents claimed that they knew how to prepare for a typhoon event. However, from our findings, their knowledge about adaptation measures was still inadequate. Most participants thought that shutting the doors and windows could reduce losses, but there were a significant proportion of participants who did not think the other measures such as keeping a watchful eye on typhoon warning, preparing emergency articles including foods and drugs, leaving the dangerous areas, keeping away from low-lying areas could work. The awareness rate of some critical issue like typhoon warning signals was low. The majority of participants claimed that they adopted coping behaviors before and during typhoon. However, only a fraction of participants actually responded when specific measures were asked. There was a gap between what they claimed and what they did. In our study, the result manifested that most residents received no typhoon preventive education, and the majority of the respondents expressed that they were willing to take part in the educational course. Previous study also suggested that compared with persons with general preparedness knowledge, persons with new knowledge and updated information were more likely to respond to typhoon effectively [16]. As a result, we suggest that the government should conduct more educational activities and make updated information available for rural areas through multiple easy-to-follow forms.

Zhejiang is one of the most economically developed coastal provinces, the insurance industry in Zhejiang province has made a great contribution in the insurance indemnity of relief work. Ningbo city had been also developed as a comprehensive insurance pilot area a few years ago [36,37]. However, the rate of disaster insurances purchased was low (26.7%), and the main reason that people did not purchase insurances included: (1) Most respondents did not know disaster insurance; (2) Not necessary to purchase; and (3) Financial reasons. In rural areas, typhoon might cause damage to their farmland or fish pond, which are the basic livelihood guarantee of the greatest majority of residents (three-quarters of the residents are farmers and fishermen). Improving publicity and promotion of affordable disaster insurance among rural residents especially farmers is also a pressing matter.

Our study found that residents with more preparation time, a higher risk perception of health damage and life threat were more likely to take measures, so did people who were scared of typhoons. In addition, our study also found that residents with a clear understanding of typhoon warning signals would be more likely to adopt measures than others. Although residents may get information from TV to know the intensity and arrival time of typhoon to take responses, a better understanding of the typhoon warning signals also contributes people to adopt effectively coping measures to some extent, especially when there is blackout caused by a typhoon. While previous studies suggested that the perception of a high risk of injury is important in individual’s evacuation decision making in the face of disaster [38,39], this survey found that residents with a higher perception of health damage and life threat would adopt less measures. The survey did not provide data that explains why and, accordingly, further research is necessary.

In general, residents in costal rural areas have a high risk perception of property damage while that of health damage and life threat need to be improved. Most people thought they were aware of how to adopt measures to reduce losses, they were in fact still less knowledgeable and their coping behaviors were inadequate. This indicates an urgent need for appropriate targeted strategies and a necessity to develop educational and other suitable information activities to eliminate the gap. Public anti-disaster capacity building is linked with a strong awareness of disaster prevention. Only when residents have fully realized the influences and harmfulness from typhoons are they able to take the corresponding preventive measures seriously. Our findings will be useful for developing strategies to make adaptations to minimize the health and property loss.

## 5. Limitations

Our study has some limitations. First, our survey was conducted 10 days after the typhoon, people’s attitude and risk perception may change slightly before and after the landfall of the typhoon, and the difference could not be compared. Typhoon may improve the perception and awareness to some extent. During our investigation, we tried to use objective indicators as many as possible in questionnaires and explicitly asked participants face-to-face about their attitude and perception before typhoon to control this bias. Secondly, there were many young farmers going out for work, and over half of the participants were older than 50, thus the results might not represent the situation in total population. However, migrant workers are also a common phenomenon in rural areas in China, thus we believe that our participants are a representative sample of those who stay in the village and the results can still provide implications of how rural communities might cope with typhoons.

## 6. Conclusions

Based on our findings, the rural residents in Zhejiang Province had a high attention on typhoon disaster prevention and a high-risk perception of property damage, while paying less attention to the risk perception of health and life risks. Most residents knew some measures to reduce losses, however the actual coping behaviors were inadequate. There was a gap between residents’ cognition or knowledge and behavior. Only a fraction of the participants received specialized education before. The popularizing rate of disaster insurance was pretty low. The government should further make strategies including educational activities, to eliminate the knowledge and behavior gap, improve typhoon preparedness, actively promote and expand the coverage of disaster insurance to rural residents.

## Figures and Tables

**Table 1:** Demographic characteristics of the participants and coping behaviors or not (N = 659).

Characteristics	Frequency	%	Adopt Behaviors ^a^		χ^2^	*p*
			Yes	No		
Gender						
Male	311	47.2	257	53	0.001	0.988
Female	348	52.8	287	59		
Age group						
14~34	110	16.7	84	25	5.542	0.236
35~44	125	19.0	102	22		
45~54	176	26.7	153	23		
55~64	151	22.9	128	23		
65~74	97	14.7	77	19		
Educational status						
Illiterate	198	30.1	172	26	9.429	0.051
Primary	165	25	123	40		
Secondary	188	28.5	159	29		
Higher secondary	77	11.7	65	11		
Post-secondary and above	31	4.7	25	6		
Occupation						
Farmer	468	71	373	95		
Fisherman	30	4.6	28	2		
Worker	45	6.8	39	2		
Student	22	3.3	17	5		
Staff	21	3.2	18	3		
Merchant	20	3.1	18	2		
Others	53	8	48	3		
Marital status						
Married	576	87.4	479	94	3.081	0.214
Single	50	7.6	37	13		
Others	33	5	23	4		

^a^ Due to the few missing value, sum of Yes or No might be not equal to total.

**Table 2:** Information sources and attitude to typhoon disaster prevention and education among rural residents in Zhejiang, China (N = 659).

Items	Frequency	%
The main ways to get information before typhoon ^a^		
Television	550	83.5
Short message service (SMS)	215	32.7
Relatives and friends	183	27.8
House-to-house notification	137	20.8
Internet	104	15.8
Broadcast	39	5.9
Newspaper	10	1.5
The most popular way to get typhoon information ^a^		
Television	506	76.8
House-to-house notification	289	43.9
Short message service (SMS)	237	36.0
Relatives and friends	131	19.9
Internet	104	15.8
Broadcast	68	10.3
Newspaper	7	1.1
Have you paid attention to typhoon disaster prevention?		
Great attention	121	18.5
Attention	489	74.8
Not sure	35	5.3
Inattention	1	0.2
Great inattention	8	1.2
Have you received any formal education of typhoon disaster prevention?		
Yes	204	31.1
No	452	68.9
Will you attend the relative training?		
Yes	548	83.7
No	107	16.3
Necessity to develop the education		
Strongly necessary	119	18.1
Necessary	472	71.6
Not sure	19	2.9
Unnecessary	46	7.0
Strongly unnecessary	3	0.5
Attitude to governmental measures like evacuation and temporary shelter		
Strongly supported	199	30.3
Supported	420	63.8
Not sure	22	3.3
Unsupported	16	2.4
Strongly unsupported	1	0.2

^a^ Percentage total may add up to more than 100% as multiple responses were permissible.

**Table 3:** Perception of risk before typhoon among rural residents in Zhejiang, China (N = 659).

Items before Typhoon	1 (%)	2 (%)	3 (%)	4 (%)	5 (%)	Mean	SD
1. What do you think the impact of this typhoon disaster?	0.3	10.1	13.2	53.0	23.4	3.89	0.886
2. Do you think this typhoon can cause property damage to you?	0.3	9.5	13.7	58.5	18.0	3.84	0.837
3. Do you think this typhoon can cause health damage to you?	3.2	27.9	18.3	46.2	4.5	3.21	1.003
4. Do you think this typhoon can cause life threat to you?	3.5	37.3	17.0	38.8	3.4	3.01	1.019
5. The degree of your fear to this typhoon?	3.2	25.9	33.5	27.6	9.8	3.15	1.016

Note: 1–5 represent the degree of very small to very big or extremely impossible to extremely possible (3 is median represent “not sure”, ≥3.65 represent high, 2.36 to 3.64 represent medium or not high).

**Table 4:** The knowledge and behaviors of typhoon disaster prevention among rural residents in Zhejiang, China (N = 659).

Knowledge and Behaviors Item	Frequency	%
Awareness of typhoon warning signal (Blue, Yellow, Orange, Red) ^a^		
Understand	78	11.9
Do not know or not understand	578	88.1
Do you know how to adopt measures for typhoon disaster prevention?		
Yes	493	74.9
No	165	25.1
Did you adopt measures before and during typhoon?		
Yes	544	82.9
No	112	17.1
Do you think the following measures can reduce losses? ^b^		
Shut the doors and windows	611	93.0
Keep a watchful eye on typhoon warning	360	54.8
Prepare emergency articles including foods and drugs	355	54.0
Leave the dangerous areas	348	53.0
Keep away from low-lying areas	317	48.2
Check the electric circuit facilities	291	44.3
Keep the vehicles	239	36.4
Environmental disinfection after typhoon	214	32.6
Stay away until it is safe to return after typhoon	211	32.1
Which measures below did you adopt this time?		
Stop all outdoor activities	434	65.9
Examine and repair the house	289	43.9
Store foods and water	277	42.0
Rush in the harvest	53	8.0
Prune the dead tree and fragile branches	47	7.1
Stay overnight at out-of-town friends’ or relatives’ house	38	5.7
Effectiveness of the preventive measures (N = 544)		
Strongly effective	56	10.3
Effective	459	84.1
Not sure	22	4.0
Ineffective	0	0
Strongly ineffective	9	1.6
Did you purchase disaster insurance?		
Yes	173	26.7
No	476	73.3
Necessity to purchase disaster insurance		
Strongly necessary	83	12.8
Necessary	422	65.0
Not sure	37	5.7
Unnecessary	100	15.4
Strongly unnecessary	7	1.1

^a^ The typhoon warning signal is a series of warning promulgated by the China Meteorological Administration (CMA) to indicate intensity of typhoon by color (blue, yellow, orange, red). The real-time warning is issued by the local government to inform people the intensity grade and arrival time of typhoon, so that people can react immediately. For example, the red warning means that typhoon will land within 6 h with an average wind speed of grade 12 or above. The blue warning means that typhoon will land within 24 h with an average wind speed of grade 6 or above; **^b^**Percentage total may add up to more than 100% as multiple responses were permissible.

**Table 5:** Factors related to adopting typhoon prevention measures among rural residents in Zhejiang, China–Complementary log–log model.

Items	Control	B	S. E	P	OR	95% CI
Understanding typhoon warning signal	Do not know	0.671	0.2095	0.001 **	1.96	1.30–2.95
Preparation time longer than two days	Two days or less	0.305	0.0971	0.002 **	1.36	1.12–1.64
Perception of property damage ^a^		0.064	0.072	0.374	1.07	0.93–1.23
Perception of health and life damage ^a^		−0.184	0.0344	<0.001 **	0.83	0.78–0.89
Fears of typhoon ^a^		0.174	0.0588	0.003 **	1.19	1.06–1.34
Attention ^a^		0.129	0.0665	0.053	1.14	0.99–1.30

^a^ Regarded as continuous variable; ** The difference was statistically significant. Note: Log likelihood = −137.076, Likelihood Ratio Chi-Square (LR Chi2) = 206.586, *p* < 0.001.

## Abbreviations

CDC	Center for Disease Control and Prevention
CMA	China Meteorological Administration
ERC	Ethics Review Committee
SMS	Short Message Service
SD	Standard Deviation
OR	Odds Ratio
CI	Confidence interval
CLL	complementary log-log
SPSS	Statistical Product and Service Solutions
